# Sex differences in the prevalence and determinants of HPV-related external genital lesions in young adults: a national cross-sectional survey in Brazil

**DOI:** 10.1186/s12879-020-05376-x

**Published:** 2020-09-18

**Authors:** Juliana Comerlato, Natália Luiza Kops, Marina Bessel, Jaqueline Driemeyer Horvath, Bruna Vieira Fernandes, Luisa Lina Villa, Flavia Moreno Alves de Souza, Gerson Fernando Mendes Pereira, Eliana Márcia Wendland

**Affiliations:** 1 Hospital Moinhos de Vento, PROADI – SUS, Ramiro Barcelos, 910, Porto Alegre, RS 90035-004 Brazil; 2 Federal University of Health Sciences of Porto Alegre, Graduate Programs in Health Sciences and Pediatrics, Porto Alegre, Brazil; 3 Hospital Moinhos de Vento, Porto Alegre, Brazil; 4 Faculdade de Medicina, University of São Paulo and Instituto do Câncer do Estado de São Paulo (ICESP), São Paulo, Brazil; 5 Department of Chronic Conditions and Sexually Transmitted Infections, Ministry of Health, Brasília, Brazil

**Keywords:** Human papillomavirus, Genital warts, HPV, Epidemiology, Prevalence in young adults

## Abstract

**Background:**

External genital lesions (EGL) are the most common sexually transmitted infections (STIs). We aimed to evaluate the prevalence, determinants and sex differences in EGL among young adults from Brazil.

**Methods:**

Overall, 7694 participants (aged 16 to 25 years) underwent an interview, genital examination and sampling for HPV genotyping.

**Results:**

The prevalence of EGL was 4.08% (234) and is more frequent in men (5.72%) than women (2.31%) (*p* <  0.001). Genital lesions were significantly associated with male sex, infection by high-risk and multiple HPV types, having more than two sexual partners in the last year, smoking status and the presence of other STI. While alcohol use was associated with a higher prevalence of EGL in women, same-sex sexual relationship increase the prevalence in men. In the EGL group, 67.79% (*p* = 0.032) were positive for HPV infection and the types HPV6 and HPV11 were the most prevalent ones.

**Conclusion:**

The prevalence of EGL in young adults was consistently high, and most cases were associated with genital HPV infection and STIs. Although men have a higher prevalence, both sexes share most genital lesion determinants. The promotion of sexual education and vaccination especially focus in young men, who are usually outside the targets of primary health care programmes, can prevent EGL in Brazilian young adults.

## Background

Human papillomavirus (HPV) lesions of the external genital region, which are commonly identified as genital warts (GW), are the most common outcome of HPV infection, followed by low-grade squamous intraepithelial lesions and cervical, penile, anal and head and neck cancers [1, 2]. The lifetime probability of acquiring HPV was estimated to be 84.6% among women and 91.3% among men [3]. Among all HPV types, HPV6 and HPV11 are the most commonly detected types responsible for external genital lesions (EGL). HPV6 and HPV11 belong to the low-risk HPV (LR-HPV) group and are known for their low association with carcinoma development [4]. Additionally, the costs associated with the diagnosis and treatment of HPV lesions reported in the international literature are substantial [5–7]. In Brazil, the healthcare costs for genital warts treatment among the sexually active population is estimated to range from $34 to $40 million over the next five years [8].

The presence of EGL is commonly associated with the presence of HPV genital infection; however, the diagnosis of HPV is often difficult, and the treatment is usually performed in a syndromic approach [9]. Additionally, 90% of individuals with genital HPV infection do not develop symptoms as numerous individual factors are associated with different outcomes [4]. Several characteristics, such as sex, race/skin colour, age at first intercourse, smoking status, the presence of other sexually transmitted infections (STI), multiple HPV infections and lifetime number of sexual partners, have been linked to the presence of genital lesions [10].

HPV immunization provides direct protection against HPV types included in the vaccine. Currently, the national HPV vaccination programme in Brazil provides the quadrivalent HPV vaccine (HPV 6/11/16/18) for girls and boys between 9 and 14 years old. Although the types commonly associated with genital lesions are included in the quadrivalent vaccine, multiple infection types can cause an increase in genital lesion incidence; therefore, it is important to understand the type distribution in the Brazilian population [11, 12].

To date, few studies investigating the association among EGL, the presence of genital HPV infection and genotype in Latin America have been conducted [13]. The currently published studies have been restricted to male individuals and include limited nationwide sampling [13–16].

Considering that data regarding the prevalence of EGL and associated factors are scarce in Brazil, the purpose of this study is to evaluate the prevalence, determinants and sex differences in EGL among sexually active young adults from Brazil.

## Methods

We used data from the POP-Brazil study, a cross-sectional, multicentric, nationwide study designed to evaluate HPV infection and its associated factors among sexually active women and men aged between 16 and 25 years in all geographic regions of Brazil [17, 18], to evaluate the prevalence of external genital lesions. In total, 7694 samples from non-vaccinated individuals from 119 primary care units were collected between September 2016 and November 2017 by trained primary health care professionals. The exclusion criteria were as follows: pregnancy, previous hysterectomy or trachelectomy, and a history of cervical intraepithelial neoplasia grade 2 or higher.

All individuals consenting to participate were invited to answer a standardized questionnaire based on validated instruments to provide information regarding sociodemographic factors and prior and current sexual health and behaviours. The presence of STIs (HIV, syphilis, gonorrhoea and/or herpes) was self-reported. Additionally, the participants were invited to undergo a rapid HIV and syphilis test. During the sampling, the presence of any lesion in the external genital region was investigated and reported by the health care professional. Genital HPV infection was assessed in cervical samples collected with a digene® HC2 DNA Collection Device (Qiagen, Hulsterweg, the Netherlands) and by supervised penile auto-collection using a digene® Female Swab Specimen Collection Kit (Qiagen). Genital (cervical or penile) samples were analysed to detect HPV infection by DNA extraction with a MagNA Pure LC 2.0 Instrument and DNA Isolation Kit III (Roche Life Science, Basel, Switzerland); the HPV detection and genotyping were conducted with a Linear Array® HPV Genotyping Test (LA HPV) (Roche Life Science, Basel, Switzerland). The LA HPV is based on PCR amplification [450 base pair (bp) fragment of the polymorphic region of the L1 gene of HPV], followed by specific hybridization and colorimetric detection according to the manufacturer’s instructions. The test can detect thirteen types of high-risk HPV (HR-HPV) (16, 18, 31, 33, 35, 39, 45, 51, 52, 56, 58, 59 and 68) and twenty-four types of LR-HPV (6, 11, 26, 40, 42, 53, 54, 55, 61, 62, 64, 66, 67, 69, 70, 71, 72, 73, 81, 82, 82v [IS39], 83, 84, and 89 [CP6108]). The test include the amplification of the human β-globin (268 bp fragment) gene was used as an internal control for sample adequacy. An AutoBlot instrument (MedTec Biolab, Hillsborough, NC, USA) was used to automate the hybridization and wash steps. The LA HPV test probes against HPV types 33, 35, 52, and 58 are cross-reactive; therefore, if the tests using these probes were positive, we performed additional analyses using a specific real-time PCR assay as previously described [19]. The samples were stored and transported to the laboratory according to the recommendations by the protocol manufacturer. All samples were processed in the same laboratory.

The data were analysed using SAS software (Statistical Analysis System, SAS Institute Inc., Cary, NC, USA) version 9.4. The descriptive data are presented as frequencies (%), means and confidence interval (CI). ANOVA, Chi-squared or Student’s t-test were used to compare the characteristics between the groups. To compare proportions, a chi-square test was used. Normal distribution was determined through the Anderson-Darling test, and Student’s t-test was applied to compare the continuous variables when appropriate. A Poisson regression with robust variance was conducted to examine the factors associated with EGL. A theoretical framework was structured as follows with the associated variables differentiated in hierarchical blocks: HPV presence (Model 1); model 1 + age (Model 2); model 2 + alcohol consumption (Model 3); model 3 + condom use, having more than 2 partners in the prior year, and STI presence (Model 4) [20]. To adjust for the distribution of the sample to the study population, we weighted the measures by the population size in each capital and sex. Therefore, all results are reported as weighted, and statistical significance is defined as *p* <  0.05.

## Results

Of the 7694 participants included in the study, the prevalence of EGL was 4.08% (95% CI: 3.22–4.94), and significant differences in prevalence were observed according to sex. Men have a higher prevalence of EGL (5.72%) than women (2.31%, *p* <  0.001).

The group of participants with lesions had a higher frequency of STIs (42.97%) than the groups of participants without lesions (12.78%; *p* <  0.001). The EGL group were also associated with current smoking (24.41%), alcohol consumption (82.68%), having two or more sexual partners in the prior year (50.68%) and having a same-sex relationship (14.87%). A higher prevalence of lesions was observed in the Southeast region (30.00%, *p* <  0.001). Significant differences were not observed in the variable “age at first sexual intercourse” between the groups [15.38 mean age in years of individuals with lesions (95% CI: 15.09–15.67) versus 15.35 without lesions (95% CI: 15.30–15.40)]. Age, race/skin colour, relationship status, and condom use were not associated with the presence of EGL (Table 1).

**Table 1:** Sociodemographic characteristics and sexual behaviour associated with external genital lesions (EGL) in Brazilian young adults

	EGL group	Without lesions	*P*-Value
Characteristics	%	%	
Age			0.091
16-17	4.56	11.11	
18-19	18.47	21.76	
20-21	20.30	22.27	
22-23	25.10	22.34	
24-25	31.57	22.51	
Race/skin colour			0.494
White	24.02	23.67	
Black	11.98	17.14	
Brown	62.08	56.64	
Other	1.92	2.55	
Relationship status			0.137
Single or without partner	33.13	24.00	
Dating or flirting	39.41	41.13	
Married or living together	27.46	34.87	
Number of sexual partners in the last year			< 0.005
< 2	49.32	67.78	
≥ 2	50.68	32.22	
Smoking status			0.039
Non-smoker	53.23	64.88	
Smoker	24.41	15.18	
Ex-smoker	22.36	19.93	
Region of Brazil			< 0.001
Midwest	27.04	11.68	
Northeast	22.26	28.51	
North	12.26	12.57	
Southeast	30.00	40.66	
South	8.44	6.58	
Alcohol consumption	82.68	71.75	0.048
Condom use	60.44	50.06	0.069
History of same-sex sexual experience	14.87	7.40	0.041
Presence of STI	42.97	12.78	< 0.001
Syphilis infection	4.54	3.86	0.708

*STI* sexually transmitted infections

**Fig. 1:**
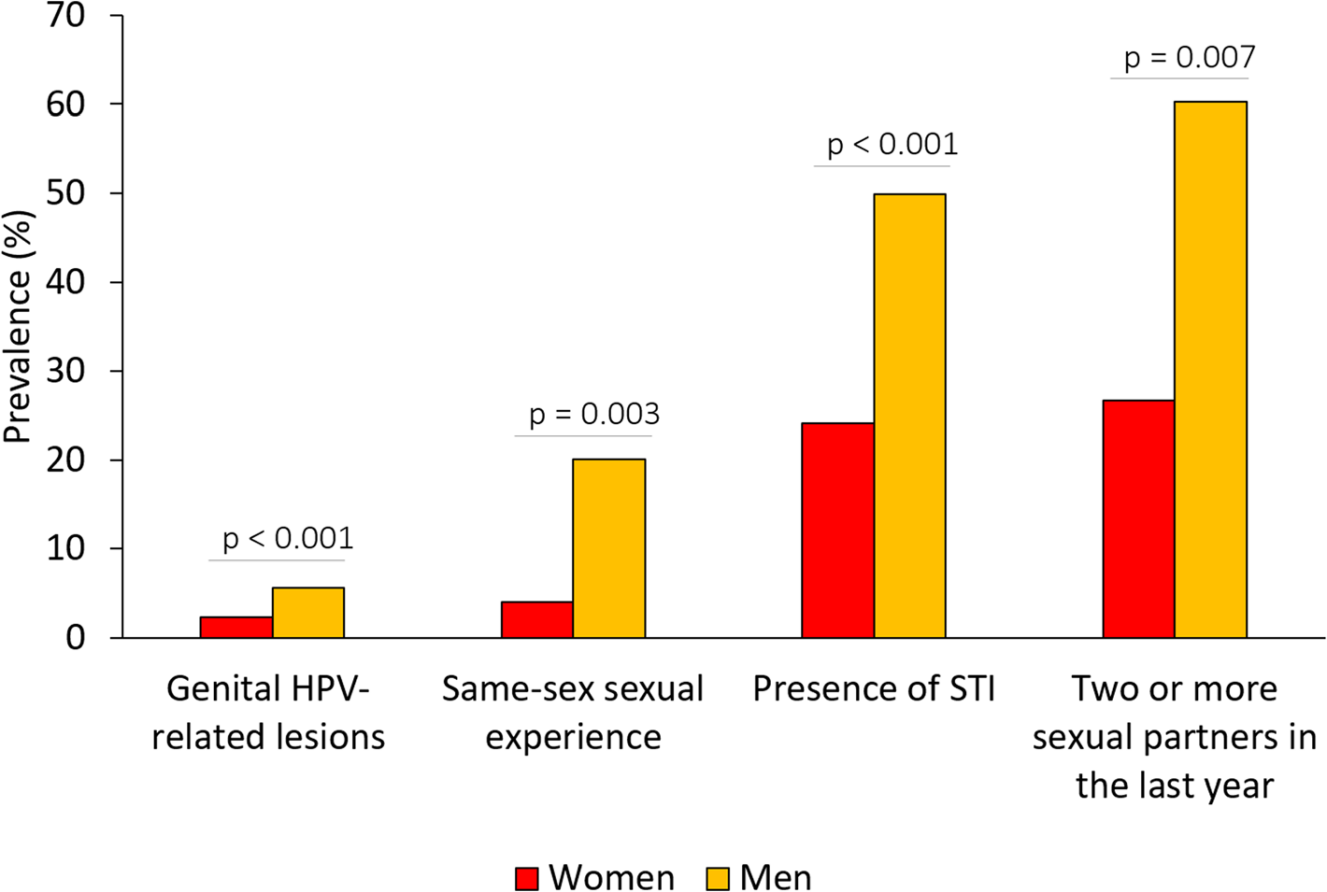
Sex differences in external genital lesions prevalence and associated characteristics

**Fig. 2:**
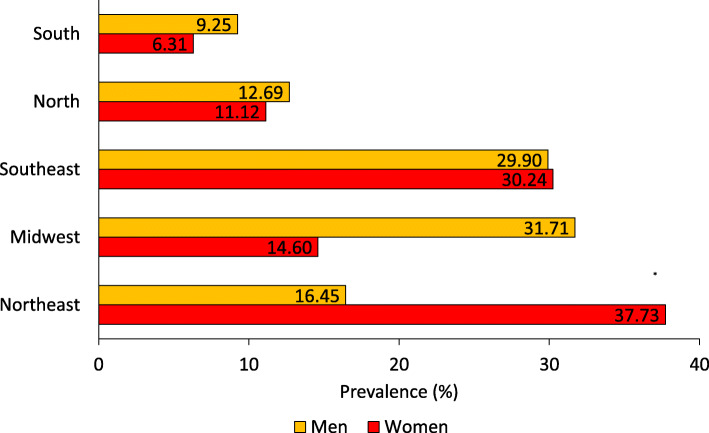
Sex distribution of external genital lesions by Brazilian geographical region. (*P value = 0.032)

The characteristics associated with the presence of EGL varied according to sex. Men had more same-sex sexual experiences, a higher number of partners in the prior year and more STIs than the women (Fig. 1). The prevalence of lesion also differed significantly by geographic region: the highest positivity in women was reported in Northeast (37.73%), and that among the men was observed in the Midwest (31.71%) (Fig. 2).

**Fig. 3:**
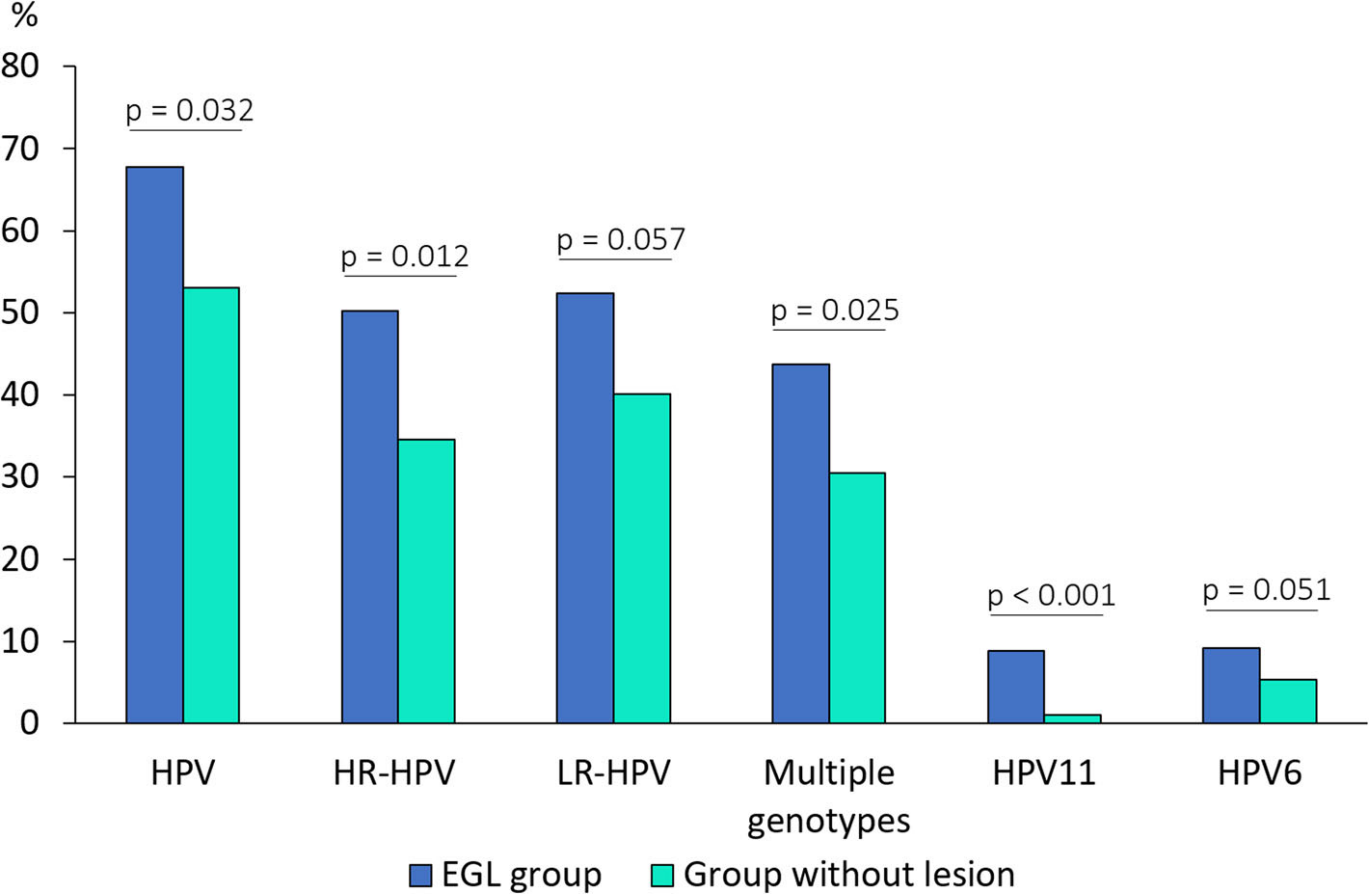
HPV DNA prevalence in participants with and without external genital lesions (EGL)

Of the 234 individuals with lesions, 195 provided valid samples for analysis (human β-globin positive); of these individuals, 67.79% (132) were positive for HPV DNA compared to 53.03% positivity among those without lesions (*p* = 0.032). The frequency of infection by HR-HPV (50.20%) and multiple genotype infection (43.71%) were higher among the people with lesions than among those without lesions (HR-HPV; 34.54%, *p* = 0.012 and multiple infection; 30.49%, *p* = 0.025). There was no statistically significant difference in LR-HPV infection between the groups (52.37% with lesions versus 40.08% without lesions, *p* = 0.057) (Fig. 3).

The most frequently identified HPV genotypes in the participants with lesions were HPV16 (12.55%), HPV6 (9.22%), HPV11 (8.82%), HPV62 (8.67%), HPV58 (8.36%), HPV52 (8.19%) and HPV51 (8.01%). By comparing the groups with and without lesions, HPV11 (8.82% versus 1.09% without lesions; *p* < 0.001) and HPV6 (9.22% versus 5.36%; *p* = 0.051) were significantly higher in the group with lesions. There was no difference in the prevalence of specific HPV types according to sex (data not shown).

When we stratified the multivariate analysis by sex, HPV infection was associated with EGL only in the women [PR 1.74 (95% CI: 1.03–2.96)]. However, HPV lost significance after incorporating STIs into the model, and no interaction was observed between HPV and STI. Alcohol consumption was independently associated with the presence of lesions in the women [PR 1.91 (95% CI: 1.09–3.33)]. HPV was not associated with lesions in men, and those with STI were 3.13 times (95% CI: 1.57–6.24) more likely to have genital lesions than non-exposed subjects (Table 2).

**Table 2:** Prevalence ratios of determinants of external genital lesions according to sex. POP-Brazil Study, 2016–2017

	Prevalence Ratio (95% CI)			
	Model 1	Model 2	Model 3	Model 4
Women				
Presence of HPV	1.87 (1.11–3.16)*	1.87 (1.10–3.19)*	1.74 (1.03–2.96)*	1.35 (0.78–2.33)
Age				
≤ 20 years		1	1	1
> 20 years		1.01 (0.61–1.67)	1.00 (0.61–1.65)	0.86 (0.52–1.43)
Alcohol consumption			2.17 (1.30–3.62)*	1.91 (1.09–3.33)*
Condom use				0.77 (0.46–1.29)
More than 2 partners in the last year				1.18 (0.67–2.06)
Presence of STI				2.03 (1.10–3.73)*
Men				
Presence of HPV	1.87 (0.87–4.03)	1.80 (0.82–3.95)	1.84 (0.83–4.07)	1.99 (0.82–4.83)
Age				
≤ 20 years		1	1	1
> 20 years		1.74 (0.84–3.59)	1.71 (0.82–3.56)	1.22 (0.56–2.64)
Alcohol consumption			1.12 (0.42–2.98)	2.53 (0.81–7.94)
Condom use				0.46 (0.21–1.01)
More than 2 partners in the last year				1.69 (0.75–3.82)
Presence of STI infection				3.13 (1.57–6.24)*

*HPV* Human Papillomavirus, *STI* Sexually Transmitted Infection, *CI* Confidence Interval, *Model 1* presence of HPV, *Model 2* model 1 and age, *Model 3* model 2 and alcohol consumption, *Model 4* model 3 and condom use, more than two partners in the last year, and presence of STI. *p < 0.05

## Discussion

We studied a large Brazilian young adult population aged 16 to 25 years and found that the prevalence of external genital lesions was 4.08%. This is the first nationwide study to assess the prevalence of EGL and its differences by sex in all five Brazilian geographical regions. In our study, the prevalence of lesions was higher in men (5.72%) than women (2.31%) and was associated with HPV infection only when STIs were not considered. The viruses commonly associated with lesions, HPV6 and HPV11, [21] were also most frequently associated with EGL in this study.

The prevalence rates found are high, similar to that reported in Mexico (5.10%), Hungary (4.03%) and the USA (4.10%), and much higher than that in most countries, such as Italy (0.05%), Canada (1.10%) [22] and Vietnam (0.20%); the peak incidence in the young population occurred between the ages of 18 and 29 years [1, 23].

Data regarding the prevalence of genital lesions in men from low/middle income countries are scarce [1, 24–26]. These studies used different epidemiological and analytical methodologies, which could explain the differences in prevalence: 5.25% in Peru [1], 1.22% in India [24] and 1.99% in Mexico [25]. The incidence reported in Mexico was 42%, and that in Brazil was 31.9% over a period of four years [26]. The higher prevalence of genital lesions in men could be explained by the higher frequency of STIs in this population [17]. A previous study performed in the women from a region on the North of Brazil associated HPV with other STIs, suggesting that individuals with HPV infection are at an increased risk of having another STI [27]. Similar results have already been reported in Seoul, Korea and Trieste, Italy [28, 29]. Additionally, STIs other than HPV or HPV co-infection, could also lead to EGL. The self-reported STIs by the participants could also include some HPV lesions, leading to a higher association with EGL.

We found a statistically significant association between alcohol consumption and the presence of EGL. An association between alcohol and HPV prevalence has been previously reported. Alcohol intake was associated with a higher positivity of HPV infection and HR-HPV types in men [30]. In women, alcohol intake was associated with a higher HPV viral load, a higher risk of cervical intraepithelial neoplasia grade 1 (CIN1), and HPV infection in female sex workers [31, 32]. The increase in genital lesions associated with alcohol could be due to an increase in the presence of HPV infection or a greater susceptibility to the development of EGL.

The rate of HPV infection in the participants with EGL (67.79%) was similar to that reported in other studies (72.3% in Brazil, 61.3% in the USA, and 61.9% in Mexico - HIM Study), but lower than the results obtained in Thailand (88.3%), Turkey (75.8%), Colombia (88.9%), China (97.0%) and Ireland (78%) [13, 14, 33–36]. The discrepancy between these results could be due to differences in the sampling techniques or methods, misdiagnoses or true differences among the populations. The LA HPV test, which is the technique chosen in this study, presents a high sensitivity (96%) and specificity (99%), and it is unlikely that variations in the genotyping test could explain the lack of positivity in 32% of individuals with lesions; however, we cannot exclude the possibility of the existence of HPV types undetectable by this technique or those individuals with low viral loads [37]. Also, a clinical diagnosis could be misleading as EGL caused by HPV clinically resemble other common genital lesions, such as those caused by molluscum contagiosum, keratosis, etc. To exclude other STIs, we asked the participants whether they have ever had a herpes virus infection and performed a syphilis rapid test. None of the participants reported genital herpes infection; however, 6.49% of the participants who presented lesions and were negative for HPV infection were positive for syphilis (data not shown).

Among the participants with genital lesions positively genotyped to HPV, 52.37% were positive for LR-HPV, and 50.20% were positive for HR-HPV. Although our study population had a much lower rate of HPV6 and/or HPV11 (24.55%) than other populations, [38, 39] we found a high frequency of HR-HPV types not commonly associated with EGL. Additionally, high rates of multiple infections were observed; this result is consistent with the data recently published by Hasanzadeh et al. (2019), who demonstrated that patients with multiple infections by LR- and HR-HPV types and those with a single infection by HR-HPV had an increased risk of developing GW [OR: 2.81; (95% CI: PR 1.21–6.55) and OR: 2.33; (95% CI: PR 1.03–5.27), respectively] [12].

Our study has both strengths and limitations. Because of the potential to report socially desirable responses or lack of knowledge, self-reported data have inherent biases that could lead to the underreporting of alcohol consumption or overreporting of STI. Additionally, we do not have enough data to address deeply some specific population as men and women who have same sex relationship. We also performed HPV testing of all the genital region in the men - including areas without lesions - while in the women, the cervix and external areas with lesions were tested. This strategy allowed for the identification of all HPV types present in the genitalia in addition to the area with EGL. This strategy could lead to an increase in other HPV genotypes not related to the lesion in the men and a decrease the types associated with lesions in the women. Additionally, the diagnosis of HPV-related lesions was performed only through visual inspection, leading to misclassification bias. Although we did not perform histological testing to confirm the clinical diagnosis, Hernandez-Suarez et al. concluded that only 19% of clinically diagnosed HPV-related lesion cases were not confirmed by microscopy [13].

Some specific populations as men who have sex with men and the impact of vaccination in this population compared to heterosexual men regarding the prevalence of EGL could not be addressed in this study and deserves further investigation as well as the impact of vaccination in EGL lesion in the Brazilian population.

## Conclusions

Our data provide important information regarding the prevalence of external genital lesions and their determinants while also exploring the differences between the sexes. The results can be used for the development of public health strategies to focus on the affected groups, such as young men, who are usually outside the targets of primary health care programmes. Furthermore, these data reinforce the importance of sexually transmitted infections prevention and the need to include boys in HPV vaccination programmes.

## Data Availability

The datasets used and/or analysed during the current study are available from the corresponding author on reasonable request.

## Abbreviations

CI: Confidence interval
EGL: External genital lesions
GW: Genital warts
HPV: Human papillomavirus
HR-HPV: High-risk HPV
LA HPV: Linear Array® HPV Genotyping Test
LR-HPV: Low-risk HPV
SAS: Statistical Analysis System
STIs: Sexually transmitted infections
